# Supplementary material for: First Total Diet Study of Aflatoxins in Singapore: Exposure Risk, High-Risk Foods, and Public Health Implications
Source: Toxins (Basel). 2025 Jun 25;17(7):324. doi: 10.3390/toxins17070324 (PMC12299484; doi:10.3390/toxins17070324)
Supplement: Supplementary file 1 [file toxins-17-00324-s001.zip › toxins-3666446-supplementary.pdf]

# Supplementary Material: First Total Diet Study of Aflatoxins in Singapore: Exposure Risk, High-Risk Foods, and Public Health Implications

**Ker Lew, Yu Lee Leyau, Ping Shen, Xin Li, Sherine Liew, Joachim Chua, Hui Yi Lim, Yuansheng Wu, Kern Rei Chng and Sheot Harn Chan**

**Table S1.** Total aflatoxin (AFT) concentrations of commonly consumed food in Singapore

| Entry | Food Category | n [a] | n+ [b] | Mean AFT [c] Concentration (μg/kg) | |
|---|---|---|---|---|---|
| | | | | MB [d] | LB – UB [e] |
| 1 | Nuts and seeds | 4 | 3 | 0.28 | 0.27 – 0.30 |
| 2 | Grains products | 26 | 2 | 0.04 | 0.01 – 0.07 |
| 3 | Rice products | 17 | 2 | 0.04 | 0.01 - 0.07 |
| 4 | Infant food | 146 | 6 | 0.03 | 0.003 - 0.06 |
| 5 | Bakery and confectionary | 18 | 1 | 0.08 | 0.05 – 0.11 |
| 6 | Other plant products | 212 | 4 | 0.04 | 0.01 – 0.07 |
| 6.1 | Vegetables | 144 | 2 | 0.05 | 0.02 – 0.08 |
| *6.11* | *Brassica vegetables* | *10* | *0* | *0.03* | *0 – 0.06* |
| *6.12* | *Fruiting vegetables* | *21* | *0* | *0.03* | *0 – 0.06* |
| *6.13* | *Fungi, seaweed* | *12* | *0* | *0.03* | *0 – 0.06* |
| *6.14* | *Leafy vegetables, herbs* | *33* | *0* | *0.03* | *0 – 0.06* |
| *6.15* | *Legumes* | *18* | *2* | *0.16* | *0.13 – 0.19* |
| *6.16* | *Root and tubers* | *22* | *0* | *0.03* | *0 – 0.06* |
| *6.17* | *Stalk, stem and bulb vegetables* | *28* | *0* | *0.03* | *0 – 0.06* |
| 6.2 | Fruits | 57 | 0 | 0.03 | 0 – 0.06 |
| 6.3 | Vegetable protein | 6 | 0 | 0.03 | 0 – 0.06 |
| 6.4 | Plant oils | 5 | 2 | 0.11 | 0.09 – 0.13 |
| 7 | Animal products | 175 | 0 | 0.03 | 0 – 0.06 |
| 7.1 | Meat products | 54 | 0 | 0.03 | 0 – 0.06 |
| 7.2 | Seafood products | 91 | 0 | 0.03 | 0 – 0.06 |
| 7.3 | Dairy products | 16 | 0 | 0.03 | 0 – 0.06 |
| 7.4 | Egg products | 12 | 0 | 0.03 | 0 – 0.06 |
| 7.5 | Animal Fats and oils | 2 | 0 | 0.03 | 0 – 0.06 |
| 8 | Sauces and condiments | 28 | 5 | 0.12 | 0.09 – 0.14 |
| 9 | Beverages | 16 | 2 | 0.05 | 0.02 – 0.08 |
| | Total | 642 | 25 | 26.55 | 7.81 – 45.49 |
| | Mean | | | 0.04 | 0.01 – 0.07 |

[a] n: number of composite samples. [b] $n^+$: number of positive samples.

[c] Total Aflatoxins (AFT): sum of Aflatoxin B1, Aflatoxin B2, Aflatoxin G1 and Aflatoxin G2.

[d] Medium bound (MD) denotes ND = LOD/2.

[e] Lower bound (LB) and upper bound (UB) denotes ND = 0 and ND = LOD respectively.

**Table S2.** Estimated Daily Intake (EDI) of Total Aflatoxins (AFT) for Singaporean consumers

| Entry | Food Category | Mean Estimated Daily Intake (EDI) of AFT [a] (ng/kg bw/day) LB – UB [b] | | | |
|---|---|---|---|---|---|
| | | Entire population | | Eaters-only | |
| | | Mean Consumers | High Consumers (95th percentile) | Mean Consumers | High Consumers (95th percentile) |
| 1 | Nuts and seeds | 0.001 – 0.001 | 0.008 – 0.009 | 0.037 – 0.042 | 0.108 – 0.125 |
| 2 | Grains products | 0.001 – 0.003 | 0.003 – 0.014 | 0.011 – 0.081 | 0.026 – 0.196 |
| 3 | Rice products | 0.001 – 0.012 | 0.007 – 0.073 | 0.013 – 0.113 | 0.028 – 0.260 |
| 4 | Infant food | 0.00001 – 0.001 | 0 – 0.003 | 0.002 – 0.035 | 0.002 – 0.040 |
| 5 | Bakery and confectionary | 0.0001 – 0.001 | 0 – 0.005 | 0.038 – 0.092 | 0.078 – 0.179 |
| 6 | Other plant products | 0.0001 – 0.002 | 0.00005 – 0.009 | 0.006 – 0.052 | 0.013 – 0.128 |
| 6.1 | Vegetables | 0.0002 – 0.002 | 0.00008 – 0.010 | 0.010 – 0.046 | 0.017 – 0.122 |
| *6.11* | *Brassica vegetables* | *0 – 0.004* | *0 – 0.017* | *0 – 0.039* | *0 – 0.108* |
| *6.12* | *Fruiting vegetables* | *0 – 0.002* | *0 – 0.009* | *0 – 0.046* | *0 – 0.139* |
| *6.13* | *Fungi, seaweed* | *0 – 0.0004* | *0 – 0.0004* | *0 – 0.040* | *0 – 0.105* |
| *6.14* | *Leafy vegetables, herbs* | *0 – 0.002* | *0 – 0.010* | *0 – 0.033* | *0 – 0.084* |
| *6.15* | *Legumes* | *0.002 – 0.003* | *0.0006 – 0.006* | *0.068 – 0.120* | *0.138 – 0.263* |
| *6.16* | *Root and tubers* | *0 – 0.004* | *0 – 0.024* | *0 – 0.050* | *0 – 0.159* |
| *6.17* | *Stalk, stem and bulb vegetables* | *0 – 0.002* | *0 – 0.006* | *0 – 0.014* | *0 – 0.046* |
| 6.2 | Fruits | 0 – 0.002 | 0 – 0.009 | 0 – 0.069 | 0 – 0.145 |
| 6.3 | Vegetable protein | 0 – 0.0002 | 0 | 0 – 0.053 | 0 – 0.129 |
| 6.4 | Plant oils | 0.0004 – 0.0007 | 0 – 0.001 | 0.021 – 0.028 | 0.067 – 0.089 |
| 7 | Animal products | 0 – 0.001 | 0 – 0.004 | 0 – 0.056 | 0 – 0.130 |
| 7.1 | Meat products | 0 – 0.001 | 0 – 0.004 | 0 – 0.070 | 0 – 0.168 |
| 7.2 | Seafood products | 0 – 0.0007 | 0 – 0.001 | 0 – 0.051 | 0 – 0.115 |
| 7.3 | Dairy products | 0 – 0.003 | 0 – 0.014 | 0 – 0.066 | 0 – 0.147 |
| 7.4 | Egg products | 0 – 0.003 | 0 – 0.015 | 0 – 0.031 | 0 – 0.072 |
| 7.5 | Animal Fats and oils | 0 – 0.0002 | 0 – 0.0006 | 0 – 0.003 | 0 – 0.007 |
| 8 | Sauces and condiments | 0.001 – 0.002 | 0.003 – 0.008 | 0.068 – 0.096 | 0.162 – 0.238 |
| 9 | Beverages | 0.0002 – 0.007 | 0 – 0.033 | 0.049 – 0.196 | 0.091 – 0.404 |
| | Total | 0.103 – 1.217 | 0.324 – 5.529 | 5.591 – 37.609 | 12.028 – 82.092 |
| | Mean | 0.0002 – 0.002 | 0.001 – 0.009 | 0.009 – 0.059 | 0.019 – 0.128 |

[a] Total Aflatoxins (AFT): sum of Aflatoxin B1, Aflatoxin B2, Aflatoxin G1 and Aflatoxin G2.

[b] Lower bound (LB) and upper bound (UB) figures denotes ND = 0 and ND = LOD respectively.

**Table S3.** Summary of Margin of Exposure (MOE) to Total Aflatoxins (AFT) for Singaporean consumers

| Entry | Food Category | Margin of Exposure (MOE) to AFT [a] LB – UB [b] | | | |
|---|---|---|---|---|---|
| | | Entire population | | Eaters-only | |
| | | Mean Consumers | High Consumers (95th percentile) | Mean Consumers | High Consumers (95th percentile) |
| 1 | Nuts and seeds | 47619 – 44321 | 5583 – 5196 | 1918 – 1785 | 779 – 725 |
| 2 | Grains products | 12850 – 8367 | 1932 – 1258 | 918 – 598 | 415 – 270 |
| 3 | Rice products | 17329 – 4541 | 2379 – 781 | 2149 – 843 | 557 – 393 |
| 4 | Infant food | 258097 – 2819 | * – 439 | 1290 – 493 | 1290 – 168 |
| 5 | Bakery and confectionary | 70833 – 12891 | * – 2750 | 248 – 248 | 122 – 122 |
| 6 | Other plant products | 7101 – 6519 | 15568 – 1674 | 167 – 153 | 87 – 80 |
| 6.1 | Vegetables | 7101 – 6519 | 15568 – 2089 | 167 – 153 | 87 – 80 |
| *6.11* | *Brassica vegetables* | ** – 24727* | ** – 4991* | ** – 2928* | ** – 1059* |
| *6.12* | *Fruiting vegetables* | ** – 21673* | ** – 4888* | ** – 501* | ** – 319* |
| *6.13* | *Fungi, seaweed* | ** – 75556* | ** – 87742* | ** – 1156* | ** – 525* |
| *6.14* | *Leafy vegetables, herbs* | ** – 15632* | ** – 2625* | ** – 1561* | ** – 918* |
| *6.15* | *Legumes* | *7101 – 6519* | *15568 – 8000* | *167 – 153* | *87 – 80* |
| *6.16* | *Root and tubers* | ** – 13600* | ** – 2089* | ** – 1504* | ** – 386* |
| *6.17* | *Stalk, stem and bulb vegetables* | ** – 40597* | ** – 12197* | ** – 3627* | ** – 863* |
| 6.2 | Fruits | 121429 – 12420 | * – 1674 | 2840 – 654 | 1141 – 279 |
| 6.3 | Vegetable protein | * – 709565 | * | * – 3199 | * – 1314 |
| 6.4 | Plant oils | 193182 – 176166 | * – 35325 | 3247 – 2961 | 1010 – 921 |
| 7 | Animal products | * – 6126 | * – 877 | * – 669 | * – 166 |
| 7.1 | Meat products | * – 20923 | * – 3109 | * – 842 | * – 407 |
| 7.2 | Seafood products | * – 21587 | * – 10112 | * – 669 | * – 166 |
| 7.3 | Dairy products | * – 6126 | * – 877 | * – 887 | * – 405 |
| 7.4 | Egg products | * – 20451 | * – 3474 | * – 3799 | * – 1617 |
| 7.5 | Animal Fats and oils | * – 906667 | * – 302222 | * – 51321 | * – 25905 |
| 8 | Sauces and condiments | 15755 – 15183 | 2768 – 2552 | 106 – 102 | **53 – 51** |
| 9 | Beverages | 110390 – 3891 | * – 654 | 327 – 258 | 171 – 135 |
| | Minimum (worst-case) | 7101 – 2819 | 1932 – 439 | 106 – 102 | 53 – 51 |

[a] Total Aflatoxins (AFT): sum of Aflatoxin B1, Aflatoxin B2, Aflatoxin G1 and Aflatoxin G2.
[b] Lower bound (LB) and upper bound (UB) figures denotes ND = 0 and ND = LOD respectively.
Note: Lowest figures were used to represent worst-case scenario.
* Unable to calculate MOE, due to either zero concentration (ND) or near zero consumption data, or small numbers of consumers-only resulting in a non-consumer at the 95th percentile position.

**Table S4.** Liver cancer potency in Singaporean from exposure to Total Aflatoxins (AFT)

| Entry | Food Category | Liver Cancer Risk from exposure to AFT [a] (Excess liver cancer Cases/100,000 Persons/year) LB - UB [b] | | | |
|---|---|---|---|---|---|
| | | Entire population | | Eaters-only | |
| | | Mean Consumers | High Consumers (95th percentile) | Mean Consumers | High Consumers (95th percentile) |
| 1 | Nuts and seeds | 0.0002 – 0.0003 | 0.002 – 0.002 | 0.006 – 0.007 | 0.015 – 0.016 |
| 2 | Grains products | 0.001 – 0.001 | 0.006 – 0.009 | 0.013 – 0.020 | 0.029 – 0.044 |
| 3 | Rice products | 0.001 – 0.003 | 0.005 – 0.015 | 0.010 – 0.014 | 0.021 – 0.030 |
| 4 | Infant food | 0 .00005– 0.004 | 0 – 0.027 | 0.009 – 0.024 | 0.009 – 0.071 |
| 5 | Bakery and confectionary | 0.0002 – 0.001 | 0 – 0.004 | 0.048 – 0.048 | 0.098 – 0.098 |
| 6 | Other plant products | 0.002 - 0.002 | 0.001 - 0.007 | 0.071 - 0.078 | 0.137 - 0.149 |
| 6.1 | Vegetables | 0.002 - 0.002 | 0.001 - 0.006 | 0.071 - 0.078 | 0.137 - 0.149 |
| *6.11* | *Brassica vegetables* | *0 - 0.0005* | *0 - 0.002* | *0 - 0.004* | *0 - 0.011* |
| *6.12* | *Fruiting vegetables* | *0 - 0.0005* | *0 - 0.002* | *0 - 0.024* | *0 - 0.037* |
| *6.13* | *Fungi, seaweed* | *0 - 0.0002* | *0 - 0.0001* | *0 - 0.010* | *0 - 0.023* |
| *6.14* | *Leafy vegetables, herbs* | *0 - 0.001* | *0 - 0.005* | *0 - 0.008* | *0 - 0.013* |
| *6.15* | *Legumes* | *0.002 - 0.002* | *0.001 - 0.001* | *0.071 - 0.078* | *0.137 - 0.149* |
| *6.16* | *Root and tubers* | *0 - 0.001* | *0 - 0.006* | *0 - 0.008* | *0 - 0.031* |
| *6.17* | *Stalk, stem and bulb vegetables* | *0 - 0.0003* | *0 - 0.001* | *0 - 0.003* | *0 - 0.014* |
| 6.2 | Fruits | 0 - 0.001 | 0 - 0.007 | 0 - 0.018 | 0 - 0.043 |
| 6.3 | Vegetable protein | 0 - 0.00002 | 0 | 0 - 0.004 | 0 - 0.009 |
| 6.4 | Plant oils | 0.0001 - 0.0001 | 0 - 0.0003 | 0.004 - 0.004 | 0.012 - 0.013 |
| 7 | Animal products | 0 - 0.002 | 0 - 0.014 | 0 - 0.018 | 0 - 0.072 |
| 7.1 | Meat products | 0 - 0.001 | 0 - 0.004 | 0 - 0.014 | 0 - 0.029 |
| 7.2 | Seafood products | 0 - 0.001 | 0 - 0.001 | 0 - 0.018 | 0 - 0.072 |
| 7.3 | Dairy products | 0 - 0.002 | 0 - 0.014 | 0 - 0.013 | 0 - 0.029 |
| 7.4 | Egg products | 0 - 0.001 | 0 - 0.003 | 0 - 0.003 | 0 - 0.007 |
| 7.5 | Animal Fats and oils | 0 - 0.00001 | 0 - 0.00004 | 0 - 0.0002 | 0 - 0.0005 |
| 8 | Sauces and condiments | 0.001 - 0.001 | 0.004 - 0.005 | 0.112 - 0.116 | 0.226 - 0.235 |
| 9 | Beverages | 0.0001 - 0.003 | 0 - 0.018 | 0.036 - 0.046 | 0.070 - 0.088 |
| | Maximum (worst-case) | 0.002 - 0.004 | 0.006 - 0.027 | 0.112 - 0.116 | 0.226 - 0.235 |

[a] Total Aflatoxins (AFT): sum of Aflatoxin B1, Aflatoxin B2, Aflatoxin G1 and Aflatoxin G2.

[b] Lower bound (LB) and upper bound (UB) figures denotes ND = 0 and ND = LOD respectively

Note: Highest figures were used to represent worst-case scenario.

**Table S5.** Hazard Quotient (HQ) of exposure to Total Aflatoxins (AFT) in Singaporean consumers.

| Entry | Food Category | Hazard Quotient of AFT [a] (LB -UB [b]) Eaters-only High consumers (95th percentile) |
|---|---|---|
| 1 | Nuts and seeds | 0.01 – 0.01 |
| 2 | Grains products | 0.02 – 0.04 |
| 3 | Rice products | 0.02 – 0.03 |
| 4 | Infant food | 0.01 – 0.06 |
| 5 | Bakery and confectionary | 0.08 – 0.08 |
| 6 | Other plant products | 0.11 – 0.13 |
| 6.1 | Vegetables | 0.11 – 0.13 |
| *6.11* | *Brassica vegetables* | *0 – 0.01* |
| *6.12* | *Fruiting vegetables* | *0 – 0.03* |
| *6.13* | *Fungi, seaweed* | *0 – 0.02* |
| *6.14* | *Leafy vegetables, herbs* | *0 – 0.01* |
| *6.15* | *Legumes* | *0.11 – 0.13* |
| *6.16* | *Root and tubers* | *0 – 0.03* |
| *6.17* | *Stalk, stem and bulb vegetables* | *0 – 0.01* |
| 6.2 | Fruits | 0 – 0.04 |
| 6.3 | Vegetable protein | 0 – 0.01 |
| 6.4 | Plant oils | 0.01 – 0.01 |
| 7 | Animal products | 0 – 0.06 |
| 7.1 | Meat products | 0 – 0.02 |
| 7.2 | Seafood products | 0 – 0.06 |
| 7.3 | Dairy products | 0 – 0.02 |
| 7.4 | Egg products | 0 – 0.01 |
| 7.5 | Animal Fats and oils | 0 – 0.0004 |
| **8** | **Sauces and condiments** | 0.19 – 0.20 |
| **9** | **Beverages** | 0.06 – 0.07 |
| | Maximum (worst-case) | 0.19 – 0.20 |

[a] Total Aflatoxins (AFT): sum of Aflatoxin B1, Aflatoxin B2, Aflatoxin G1 and Aflatoxin G2.

[b] Lower bound (LB) and upper bound (UB) figures denotes ND = 0 and ND = LOD respectively.

Note: Highest figures were used to represent worst-case scenario.

Tolerable daily intake (TDI) of 0.017 μg/kg bw/day (lower reference point) was used as reference value for the assessment of immune impairment.
